# MiR-191-5p alleviates microglial cell injury by targeting Map3k12 (mitogen-activated protein kinase kinase kinase 12) to inhibit the MAPK (mitogen-activated protein kinase) signaling pathway in Alzheimer’s disease

**DOI:** 10.1080/21655979.2021.2008638

**Published:** 2021-12-22

**Authors:** Wenjun Wan, Ganzhe Liu, Xia Li, Yu Liu, Ying Wang, Haisong Pan, Jun Hu

**Affiliations:** a Department of Rehabilitation Medicine, Wuhan Central Hospital Affiliated to Tongji Medical College of Huazhong University of Science and Technology, Wuhan, Hubei, China; b Department of Neurology, Wuhan Central Hospital Affiliated to Tongji Medical College of Huazhong University of Science and Technology, Wuhan, Hubei, China; c Department of Ultrasound Imaging, Hubei Provincial Hospital of Traditional Chinese Medicine, Wuhan, Hubei, China; d Department of Radiology, Hubei Provincial Hospital of Traditional Chinese Medicine, Wuhan, Hubei, China

**Keywords:** Alzheimer’s disease, miR-191-5p, Map3k12, microglia, Mapk signaling

## Abstract

Alzheimer’s disease (AD) is a progressive neurodegenerative disease. Multiple reports have elucidated that microRNAs are promising biomarkers for AD diagnosis and treatment. Herein, the effect of miR-191-5p on microglial cell injury and the underlying mechanism were explored. APP/PS1 transgenic mice were utilized to establish mouse model of AD. Amyloid-β protein 1–42 (Aβ1-42)-treated microglia were applied to establish *in vitro* cell model of AD. MiR-191-5p expression in hippocampus and microglia was measured by reverse transcription quantitative polymerase chain reaction. The viability and apoptosis of microglia were evaluated by Cell Counting Kit-8 assays and flow cytometry analyses, respectively. The binding relationship between miR-191-5p and its downstream target mitogen-activated protein kinase kinase kinase 12 (Map3k12) was determined by luciferase reporter assays. Pathological degeneration of hippocampus was tested using hematoxylin-eosin staining and Nissl staining. Aβ expression in hippocampus was examined via immunohistochemistry. In this study, miR-191-5p was downregulated in Aβ1-42-stimulated microglia and hippocampal tissues of APP/PS1 mice. MiR-191-5p overexpression facilitated cell viability and inhibited apoptosis rate of Aβ1-42-treated microglia. Mechanically, miR-191-5p targeted Map3k12 3ʹ-untranslated region to downregulate Map3k12 expression. MiR-191-5p inhibited Aβ1-42-induced microglial cell injury and inactivated the MAPK signaling by downregulating Map3k12. Overall, miR-191-5p alleviated Aβ1-42-induced microglia cell injury by targeting Map3k12 to inhibit the MAPK signaling pathway in microglia.

## Introduction

Alzheimer’s disease (AD) is a progressive degenerative disease of the central nervous system (CNS) and is the primary contributor of senile dementia [1]. According to World Alzheimer Report, 46.8 million people are living with dementia in 2015 and people suffered from AD worldwide is predicted to be 131.5 million by 2050 [2]. AD is featured with neuronal death, intraneuronal neurofibrillary tangles, and neuritic senile plaques [3,4]. Clinically, the hallmarks of AD include learning and memory impairments, aphasia, apraxia, agnosia, deficits of visuospatial skills, executive dysfunction and changes in personality and behaviors [5,6]. At present, AD cannot be cured completely and the main drugs for AD clinical treatment are N‑methyl‑d‑aspartate (NMDA) receptors and acetylcholinesterase inhibitors [7]. Moreover, histological examination at autopsy is the only definitive diagnosis for AD and two-thirds of patients with AD cannot be diagnosed timely [8]. Therefore, finding effective therapeutical approaches for clinical diagnosis and treatment of AD is of great importance.

Hippocampus is a part of the limbic system between medial temporal lobe and thalamus, which is responsible for transformation, storage and localization of long-term memory [9]. Microglia are immune cells of the CNS that play a key role in injury response, pathogen defense, and maintenance of CNS tissues [10]. Amyloid-β protein (Aβ) is the main component of senile plaque, one of histopathological hallmarks of AD. Aβ is formed by sequential cleavage of amyloid precursor protein by β-secretase (BACE1) and γ-secretase, and mainly includes Aβ40 and Aβ42, of which Aβ42 possesses higher cytotoxicity [11,12]. With strong phagocytic capacity, microglia can clear Aβ in the brain [13]. However, Aβ deposition induces inflammatory response of microglia, which subsequently accelerating microglial cell apoptosis [14]. Moreover, Aβ accumulation increases Ca^2+^ concentration and activates protein kinases in nerve cells, resulting in abnormal phosphorylation of Tau protein [15]. Aggregation of highly phosphorylated Tau protein contributes to neuronal loss and the enlargement of neurofibrillary tangles [16].

MicroRNAs (miRNAs) are small noncoding RNAs (18–25 nucleotides) found in a variety of organisms that significantly reduce the expression of target genes by binding to the 3ʹ-untranslated region (3ʹ-UTR) of the target messenger RNAs [17]. Accumulating studies have revealed that miRNAs can act as potential biomarkers in AD. For example, miR-338-5p overexpression decelerates neuron loss and mitigates cognitive dysfunction in APP/PS1 mice via directly targeting B-cell lymphoma 2 like 11 (BCL2L11) [18]. MiR-34 c targets synaptotagmin 1 through the ROS-JNK-p53 pathway to mediate synaptic deficits in AD [19]. MiR-124 is significantly elevated in the hippocampal tissues of Tg2576 mice, and suppression of miR-124 reverses memory deficits and synaptic failure by interacting with protein tyrosine phosphatase non-receptor type 1 (PTPN1) in AD development [20]. Particularly, miR-191-5p is an important regulator in attention-deficit/hyperactivity disorder [21] and multiple sclerosis [22]. Furthermore, miR-191-5p has been reported to be downregulated in AD [23] and is regarded as a serum biomarker for AD [24]. However, the pathological effects of miR-191-5p and its possible molecular mechanisms in AD are still poorly investigated.

Mitogen-activated protein kinases (MAPKs) are serine-threonine kinases that are widely existed in CNS and can mediate intracellular signaling associated with variety various cellular activities, including cell transformation, apoptosis, differentiation and proliferation [25]. MAPK family consists of c-Jun NH2-terminal kinase (JNK), extracellular signal-regulated kinase (ERK) and p38 [26]. Neuron apoptosis in AD has been demonstrated to be mediated by the MAPK pathway [27]. p38 can regulate apoptosis of neural and non-neuronal cells through numerous mechanisms, including elevation of TNF-α and c-myc levels, activation of caspase-3, Bax transposition, activation of c-JUN and c-fos, as well as phosphorylation of P53 [28–30]. The ERK pathway facilitates the migration and activation of neuros, playing a pivotal role in memory and synaptic plasticity [31]. Therefore, inhibiting neuron apoptosis by regulating the MAPK signaling may offer a potential avenue for AD treatment.

In this study, the role of miR-191-5p in AD progression was preliminarily investigated. We hypothesized that miR-191-5p might affect microglial cell injury by regulating downstream genes. Amyloid precursor protein/presenilin 1 (APP/PS1) mouse model of AD was established to explore miR-191-5p expression *in vivo*. The biological functions and underlying mechanism of miR-191-5p were investigated by establishing *in vitro* cell model of AD using amyloid-β protein 1–42 (Aβ1-42)-treated microglia. Our study may provide novel insight into therapeutic targets for AD treatment.

## Material and methods

### Bioinformatic analysis

Potential target genes of miR-191-5p were identified using miRDB (http://www.mirdb.org/) with the screening condition of target score > 85. The binding site between miR-191-5p and mitogen-activated protein kinase kinase kinase 12 (Map3k12) 3ʹ-UTR was predicted by Targetscan (http://www.targetscan.org/).

### Experimental animals

A total of 24 male APP/PS1 mice (25 ± 2 g, 4 months old) and 8 wild-type (WT) C57BL/6 J female mice (25 ± 2 g, 4 months old) were purchased from Beijing HFK Bioscience Co., Ltd (Beijing, China). All mice were given free access to water and food and housed under a 12-hour light/dark cycle. All animal experiments were performed following the ethical requirements approved by the Ethics Committee of Hubei Provincial Hospital of Traditional Chinese Medicine (Hubei, China).

### Animal grouping

APP/PS1 mice were utilized as the model group and C57BL/6 J mice were utilized as control group. All mice were anaesthetized using 3% pentobarbital sodium (30 mg/kg) (Sigma-Aldrich, St. Louis, MO, USA) via intraperitoneal injection [32]. After anesthetization, mice were decapitated and the hippocampi were stripped. A part of hippocampal tissues was fixed with 4% paraformaldehyde, dehydrated, paraffin-embedded and sliced up for histological observation.

### Hematoxylin-eosin (H&E) staining

Paraffin-embedded hippocampal sections were stained with hematoxylin-eosin solution (Sigma-Aldrich) according to the manufacturer’s instructions. To assess mean microvascular density, a light microscope (Olympus, Tokyo, Japan) was applied to determine the number of microvessels per unit area (/mm^2^) [33].

### Nissl staining

After dewaxing and rehydration, paraffin-embedded hippocampal sections were stained with Nissl staining solution (Linmei Biotechnology, Hefei, China) for 30 min at 60°C. Sections were then subjected to dehydration with anhydrous ethanol, made transparent with xylene, and sealed with neutral gum [33]. The morphology of Nissl bodies in hippocampus was observed under a light microscope (Olympus).

### Immunohistochemistry

Hippocampi sections were dewaxed in xylene and rehydrated by gradient ethanol. After washing, the sections were sealed with 3% (v/v) H_2_O_2_ and treated with sodium citrate buffer (10.2 mM) at 95°C for 20 min. After sealing with 10% (w/v) BSA in phosphate buffered saline for 10 min, the sections were incubated with anti-Aβ (Abcam, Cambridge, MA, USA) at 4°C overnight according to the manufacturer’s instructions. At last, hippocampus sections were incubated with HRP-conjugated secondary antibody and counterstained with hematoxylin. Images of hippocampus were obtained by DP2-TWAN image acquisition system (Olympus) and analyzed by Image-Pro Plus software (Media Cybernetics, USA) for Aβ quantification [33].

### Cell culture

Primary microglia were isolated by mild trypsinization from the cortex of newborn (postnatal day 0–2) C57BL/6 J mice (Beijing HFK Bioscience) as previously described [34]. In brief, the cortex was shredded and digested into a single cell suspension which was incubated in Dulbecco’s Modified Eagle Medium/Nutrient Mixture F-12 (DMEM/F-12; Gibco BRL, Grand Island, NY, USA) supplemented with 10% fetal bovine serum (Gibco) and 100 U/ml 1% streptomycin/penicillin (Gibco). After incubation for 15 d, mixed glial cells were shaken at 37°C for 2 h. Separated microglial cells were collected for the subsequent experiments.

### Cell treatment

The Aβ used in this study was human Aβ1-42 (China Peptides, Shanghai, China). The Aβ powder was dissolved, incubated with dimethyl sulfoxide (Gibco) and then diluted to a stock solution with phosphate buffered saline (Boster Biological Technology, Wuhan, China) [35]. After cell culture, microglial cells were treated with 10 μM Aβ1-42 for 24 h [35]. Aβ1-42-stimulated microglial cells served as the Aβ1-42 group. Microglial cells without Aβ1-42 treatment acted as the control (Con) group.

### Cell transfection

MiR-191-5p mimics were used to overexpress miR-191-5p with negative control (NC) mimics as the control. Full sequence of Map3k12 was subcloned into the pcDNA3.1 vector to elevate Map3k12 expression with empty pcDNA3.1 vector as a negative control. All plasmids and vectors were purchased from GenePharma (Shanghai, China) and transfected into microglia using Lipofectamine 2000 (Invitrogen, Carlsbad, CA, USA) [36]. The transfection efficiency was examined using RT-qPCR after 48 h.

### Cell counting Kit-8 (CCK-8) assay

The viability of microglial cells was detected by CCK-8 assay [37]. Microglial cells with or without Aβ1-42 treatment and miR-191-5p mimics/NC mimics transfection were seeded into 96-well plate at a density of 5 × 10^3^ cells/well. After that, Cell Counting Kit-8 (CCK-8) solution (10 μL; Yeasen, Shanghai, China) was supplemented to the culture plate and co-incubated with the cells for 4 h at 37°C. The optical density of each well at 450 nm wavelength was examined using a Microplate Reader (Multiscan EX; Labsystems, Helsinki, Finland). All experiments were conducted in triplicate.

### Flow cytometry analysis

The apoptosis of microglia was evaluated using an Annexin V-FITC/PI apoptosis detection kit (BD Biosciences, CA, USA) via flow cytometry analysis [38]. After washed with phosphate buffered saline, microglial cells with indicate treatments were resuspended in binding buffer and then stained with Annexin V-FITC (5 µL; 10 min) and propidium iodide (PI) (5 µL; 5 min) at room temperature in the darkness according to the manufacturer’s instructions. The apoptosis rate of stained microglia was analyzed by a flow cytometry (FACScan, BD Biosciences) using Cell Quest Pro software (Beckman Coulter, CA, USA). All experiments were repeated three times.

### Reverse transcription quantitative polymerase chain reaction (RT-qPCR)

Total RNA was isolated from microglial cells and hippocampus tissues of mice using TRIzol reagent (Invitrogen) [39]. Then, extracted RNA was reverse transcribed to complementary DNA using First Strand cDNA synthesis kit (Roche, Switzerland). PCR was performed using the PrimeScript RT reagent Kit (Invitrogen) on IQ5 real-time PCR system (Bio-Rad, USA) following the manufacturer’s protocols. The expression levels of miR-191-5p and Map3k12 were analyzed using the 2^−ΔΔCt^ method and normalized to U6 and GAPDH respectively. Sequences of primers were listed in Table 1.

**Table 1.** Sequences of primers used for reverse transcription-quantitative PCR

Gene	Sequence (5ʹ→3ʹ)
miR-191-5p forward	ACACTCCAGCTGGGCAACGGAATCCCAAAAG
miR-191-5p reverse	TGGTGTCGTGGAGTCG
Taf5 forward	AGACCATGCTGGACTTTCGG
Taf5 reverse	GTGAGGCCCTGGTAAGCATT
Neurl4 forward	CTTGCCTCCGGGTAAAGAGG
Neurl4 reverse	CTGGGGAATGCAAGGGGAAT
Chmp5 forward	GGATGAATTTGGACTGCCGC
Chmp5 reverse	AATCTGGTTGGCAGATCGGG
Sall1 forward	CCGGGAATGTCCAAGCTGAT
Sall1 reverse	GGGGTTGGCAGATGTTCGTA
Tjp1 forward	CGTTCCGGGGAAGTTACGTG
Tjp1 reverse	TGGAGGTTTCCCCACTCTGA
Map3k12 forward	CCGTACACCTGAGTTCCACA
Map3k12 reverse	ATCATTGTCCAGACAGGGCG
Wiz forward	CAGCGACGGCCCTATGAAG
Wiz reverse	GCATGGTGAGAGGTAAGCGT
GAPDH forward	ACCCAGAAGACTGTGGATGG
GAPDH reverse	CACATTGGGGGTAGGAACAC
U6 forward	CTCGCTTCGGCAGCACA
U6 reverse	AACGCTTCACGAATTTGCGT

### Western blotting

Radioimmunoprecipitation Assay Lysis Buffer (Beyotime, Nanjing, China) was used to extract proteins from microglial cells. Bicinchoninic Acid Protein Assay Kit (Thermo Fisher Scientific, Waltham, MA, USA) was applied to determine the concentration of isolated proteins. Protein samples were loaded at 12% sodium dodecyl sulfate polyacrylamide gel electrophoresis and then transferred onto polyvinylidene fluoride membranes (Millipore, USA). Subsequently, the membranes were incubated with primary antibodies at 4°C overnight, which included anti-Map3k12 (JK221305, Shanghai JingKang Bioengineering CO., LTD., Shanghai, China), anti-BACE1 (ab183612, 1:1000, Abcam), anti-Tau-5 (ab80579, 1:1000, Abcam), anti-p-ERK1/2 (ab278538, 1:100, Abcam), anti-ERK1/2 (ab17942, 1:1000, Abcam), anti-p-p38 (ab195049, 1:1000, Abcam), anti-p38 (ab31828, 1:1000, Abcam), and GAPDH (ab8245, 1:1000, Abcam). Then, the membranes were incubated with secondary antibodies at room temperature for 2 h. The protein bands were imaged by enhanced chemiluminescence reagent (Bio-Rad) and analyzed by ImageJ software (National Institutes of Health, Bethesda, MA, USA) [40].

### Luciferase reporter assay

The binding site between miR-191-5p and Map3k12 was predicted with TargetScan website. The wild type (Wt) or mutant (Mut) of Map3k12 3ʹ-UTR containing the binding site of miR-191-5p was subcloned into the pmirGLO vector (Promega, Madison, WI, USA) to generate Map3k12 3ʹ-UTR Wt/Mut reporter [41]. Next, pmirGLO vector carrying Map3k12 3ʹ-UTR Wt/Mut were cotransfected with miR-191-5p mimics or NC mimics into microglial cells utilizing Lipofectamine 2000 (Invitrogen) according to the manufacturer’s recommendations. Luciferase Reporter Assay System (Promega) was applied to examine luciferase activity after 48 h of co-transfection.

### Statistical analysis

GraphPad Prism 6.0. (GraphPad Software, San Diego, CA, USA) and SPSS 19.0 (SPSS Inc, Chicago, IL, USA) were used for statistical analysis [42]. Data in this study are exhibited as the mean ± standard deviation. Statistical comparisons between two groups were determined by Student’s *t*-test and statistical comparisons among multiple groups were tested by one-way ANOVA. A value was considered statistically significant if *p* < 0.05.

## Results

miRNAs are promising biomarkers for AD diagnosis and treatment. miR-191-5p was previously found to be downregulated in AD patients but its specific role in AD still unclear. Herein, we hypothesized that miR-191-5p might affect microglial cell injury by regulating downstream genes. The results revealed that miR-191-5p was downregulated in the hippocampal tissues of APP/PS1 mice and Aβ1-42-treated microglial cells. Overexpressing miR-191-5p mitigated Aβ1-42-induced cell injury. Mechanistically, miR-191-5p targeted Map3k12 to inactivate the MAPK signaling. Thus, we concluded that miR-191-5p alleviates microglial cell injury by targeting Map3k12/MAPK signaling pathway.

### MiR-191-5p is downregulated in the hippocampal tissues of APP/PS1 mice

**Figure 1.**
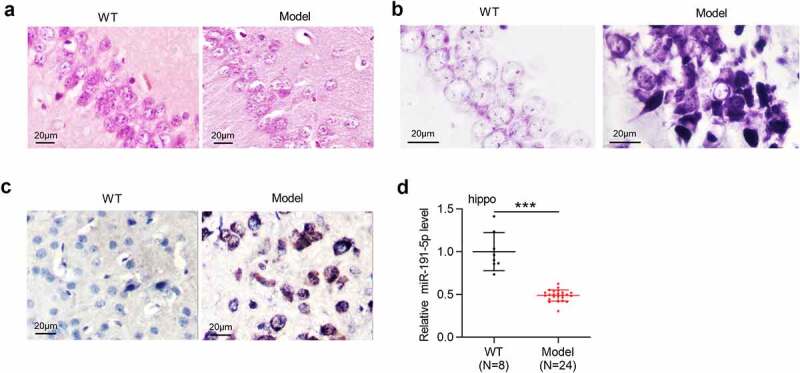
**MiR-191-5p is downregulated in the hippocampal tissues of APP/PS1 mice**. (a-b) Pathological changes of the hippocampus in C57BL/6 J mice and APP/PS1 mice were assessed by H&E staining and Nissl staining. (c) Aβ-positive granules in the hippocampal tissues of C57BL/6 J mice and APP/PS1 mice were determined by immunohistochemical staining. (b) MiR-191-5p expression in the hippocampal tissues of C57BL/6 J mice (n = 8) and APP/PS1 mice (n = 24) was detected by RT-qPCR. ***p < 0.001

Pathological degeneration of hippocampus was evaluated by H&E staining and Nissl staining. According to the results from H&E staining, the Model group showed decreased neuron number and lower neuronal cell density in the hippocampus compared with the WT group (Figure 1a). As Nissl staining demonstrated, the Model group exhibited the loss of Nissl bodies in hippocampal neurons, swollen and disrupted neurons, and disordered cell arrangement compared with the WT group (Figure 1b). Aβ-positive areas in the hippocampus were determined by immunohistochemical staining, which showed that Aβ-positive granules in the Model group were more than that in the WT group (Figure 1c). APP/PS1 mice presented obvious pathological degeneration of hippocampus, indicating that mouse model of AD were successfully established. RT-qPCR was performed to detect miR-191-5p expression in hippocampal tissues of WT mice and model mice. As Figure 1d demonstrated, miR-191-5p was downregulated in the hippocampal tissues of APP/PS1 mice compared with that in tissues of WT mice, suggesting that miR-191-5p may participate in AD progression.

### MiR-191-5p overexpression relieves Aβ1-42-induced microglial cell injury

**Figure 2.**
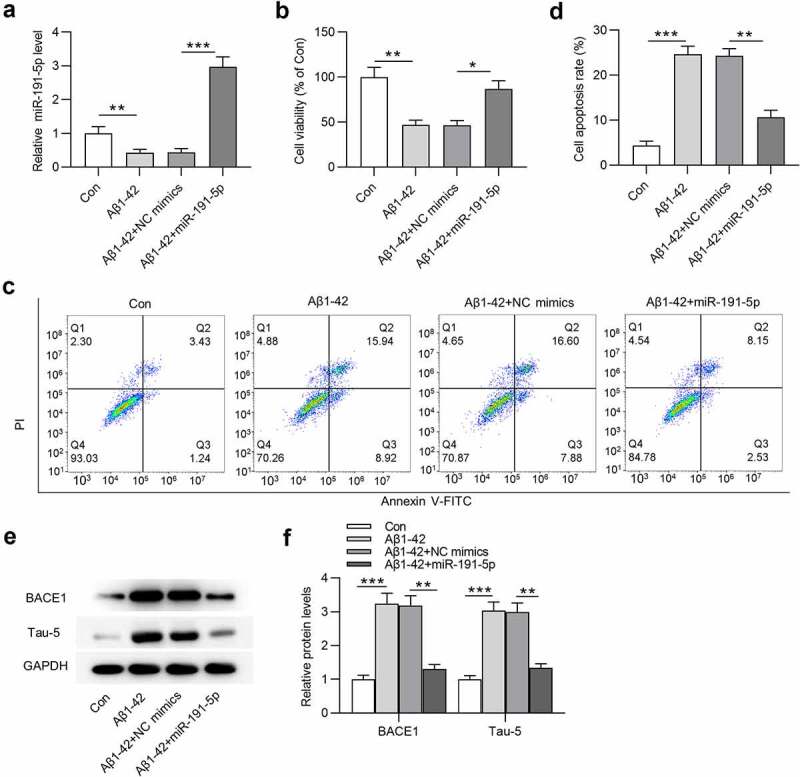
**MiR-191-5p overexpression alleviates Aβ1-42-induced microglial cell injury**. (a) MiR-191-5p expression in microglia treated with or without Aβ1-42, treated with Aβ1-42 and transfected with NC mimics, treated with Aβ1-42 and transfected with miR-191-5p mimics was measured by RT-qPCR. (b) Viability of microglial cells in the above four groups (Con, Aβ1-42, Aβ1-42+ NC mimics, Aβ1-42+ miR-191-5p) was assessed by CCK-8 assays. (c-d) Apoptosis of microglial cells in the above four groups was detected by flow cytometry analyses. (e-f) Western blotting was performed to assess protein levels of BACE1 and Tau-5 in microglial cells in the above four groups. *p < 0.05, **p < 0.01, ***p < 0.001

To establish *in vitro* cell model of AD, Aβ1-42 was utilized to treat microglia. MiR-191-5p was observed to be downregulated in Aβ1-42-treated microglia compared with that in the control group (Figure 2a). After transfection with miR-191-5p mimics, miR-191-5p expression was increased in Aβ1-42-stimulated microglial cells compared with that in cells treated with Aβ1-42 and transfected with NC mimics (Figure 2a). CCK-8 assays revealed that cell viability was decreased after Aβ1-42 treatment and miR-191-5p overexpression offset Aβ1-42-induced decrease in the viability of microglia (Figure 2b). As shown by flow cytometry analyses, miR-191-5p elevation attenuated the promoting effects of Aβ1-42 on apoptosis rate of microglial cells (Figure 2c-d). Western blotting was performed to examine protein levels of key factors (BACE1 and Tau-5) implicated with AD. The increase in BACE1 and Tau-5 protein levels caused by Aβ1-42 administration in microglial cells was counteracted by miR-191-5p upregulation (Figure 2e-f). In summary, miR-191-5p overexpression relieves Aβ1-42-induced microglial cell injury.

### MiR-191-5p targets Map3k12

**Figure 3.**
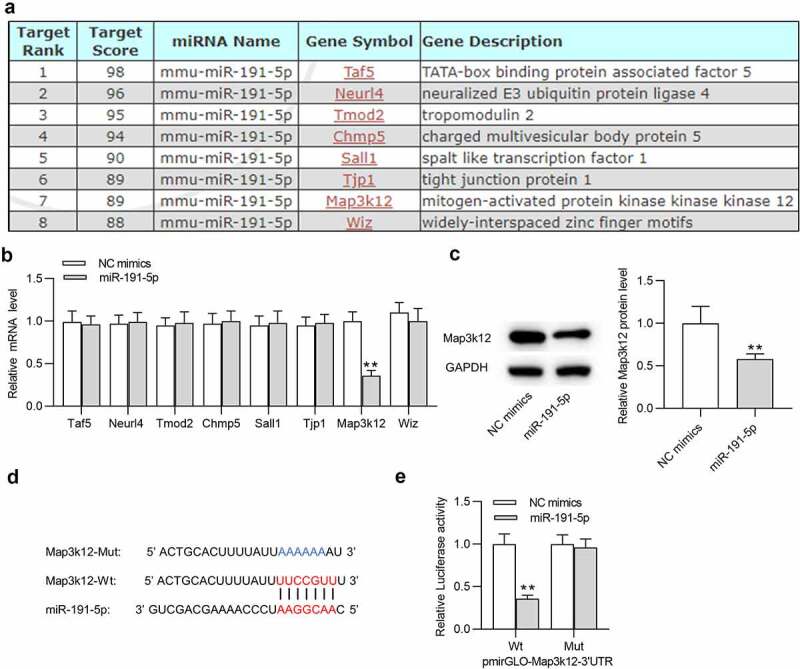
**MiR-191-5p targets Map3k12**. (a) Potential target genes (Taf5, Neurl4, Tmod2, Chmp5, Sall1, Tjp1, Map3k12 and Wiz) of miR-191-5p were predicted by miRDB with the screening condition of target score > 85. (b) The mRNA levels of potential target genes (Taf5, Neurl4, Tmod2, Chmp5, Sall1, Tjp1, Map3k12 and Wiz) of miR-191-5p in microglial cells after transfection of NC mimics or miR-191-5p mimics were examined by RT-qPCR. (c) Map3k12 protein level in microglial cells after transfection of NC mimics or miR-191-5p mimics was detected by Western blotting. (d) A binding site between miR-191-5p and Map3k12 3ʹ-UTR was predicted Targetscan. (e) Luciferase reporter assay was performed to confirm the binding relationship between miR-191-5p and Map3k12. **p < 0.01

To investigate the mechanism of miR-191-5p in microglial cells, potential target genes (Taf5, Neurl4, Tmod2, Chmp5, Sall1, Tjp1, Map3k12 and Wiz) of miR-191-5p were predicted by miRDB with the screening condition of target score > 85 (Figure 3a). RT-qPCR was performed to examine expression levels of these target genes in microglia transfected with miR-191-5p mimics. After overexpressing miR-191-5p, only Map3k12 was significantly downregulated in microglia among these candidate mRNAs (Figure 3b). Thus, miR-191-5p was selected for subsequent experiments. Overexpressing miR-191-5p also decreased Map3k12 protein level in microglia (Figure 3c). Based on Targetscan website, a binding site between miR-191-5p and Map3k12 3ʹ-UTR was predicted (Figure 3d). Luciferase reporter assays suggested that miR-191-5p upregulation markedly decreased the luciferase activity of Map3k12 3ʹ-UTR Wt rather than that of Map3k12 3ʹ-UTR Mut in microglial cells (Figure 3e). The results confirmed the binding relationship between miR-191-5p and Map3k12 3ʹ-UTR. Overall, miR-191-5p directly targets Map3k12 3ʹ-UTR in microglia.

### MiR-191-5p alleviates Aβ1-42-induced microglial cell injury by downregulating Map3k12

**Figure 4.**
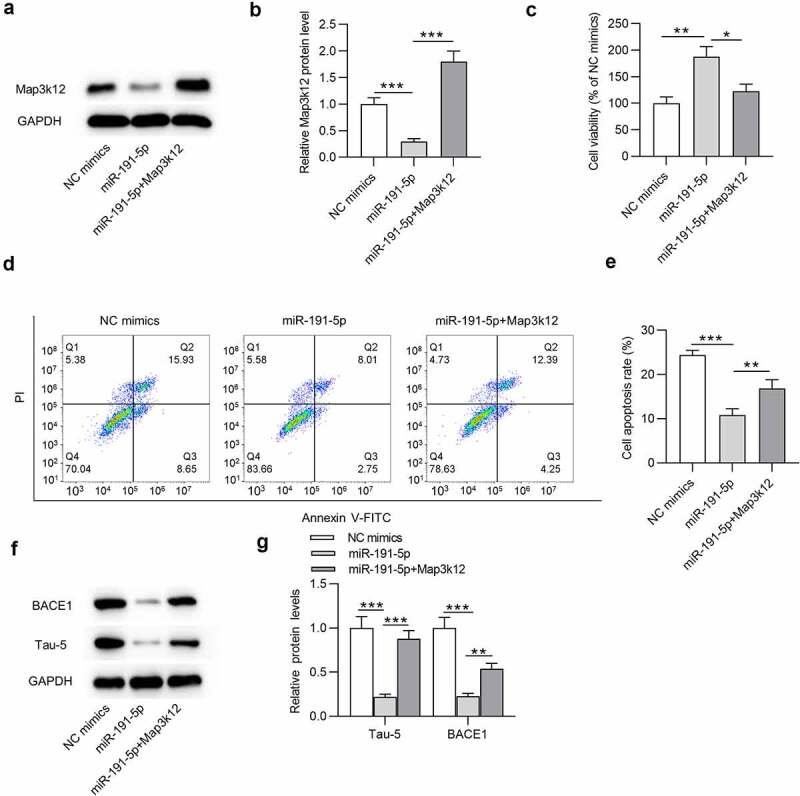
**MiR-191-5p alleviates Aβ1-42-induced microglial cell injury by downregulating Map3k12**. (a-b) Map3k12 protein level in Aβ1-42-treated microglia transfected with NC mimics, miR-191-5p mimics, or cotransfected with miR-191-5p mimics and pcDNA3.1/Map3k12 was assessed by Western blotting. (c) Viability of Aβ1-42-stimulated microglia in above three groups (NC mimics, miR-191-5p, miR-191-5p+Map3k12) was measured by CCK-8 assays. (d-e) Apoptosis of Aβ1-42-treated microglial cells in above three groups was detected by flow cytometry analyses. (f-g) The protein levels of BACE1 and Tau-5 in Aβ1-42-treated microglia from the three groups were evaluated by Western blotting. *p < 0.05, **p < 0.01, ***p < 0.001

To explore the role of the miR-191-5p/Map3k12 axis in Aβ1-42-administrated microglial cells, following experiments were performed. Map3k12 protein level was downregulated in miR-191-5p mimics group compared with that in NC mimics group in Aβ1-42-treated microglia (Figure 4a-b). Additionally, after co-transfection of miR-191-5p mimics and pcDNA3.1-Map3k12, the expression of Map3k12 was increased compared with that in miR-191-5p mimics group (Figure 4a-b). According to the results from CCK-8 assays, miR-191-5p upregulation increased the viability of Aβ1-42-treated microglial cells compared with NC mimics, and the increase in cell viability mediated by overexpressed miR-191-5p was counteracted by Map3k12 overexpression (Figure 4c). Moreover, compared with the apoptosis rate of Aβ1-42-stimulated microglia in NC mimics group, that in miR-191-5p mimics group was significantly reduced (Figure 4d-e). However, overexpressing Map3k12 rescued the decrease in cell apoptosis induced by overexpressed miR-191-5p (Figure 4d-e). Furthermore, overexpressed miR-191-5p reduced protein levels of BACE1 and Tau-5 in cells compared with the NC mimics group (Figure 4f-g). Additionally, reduction of BACE1 and Tau-5 protein levels caused by miR-191-5p elevation in microglia under Aβ1-42 treatment was mitigated by Map3k12 upregulation (Figure 4f-g). Taken together, Map3k12 elevation offsets the effects of miR-191-5p upregulation on cell viability, cell apoptosis, and BACE1 and Tau-5 protein levels in microglia under Aβ1-42 condition, indicating that miR-191-5p alleviates Aβ1-42-administrated microglial cell injury by downregulating Map3k12.

### MiR-191-5p inactivates the MAPK signaling by targeting Map3k12

**Figure 5.**
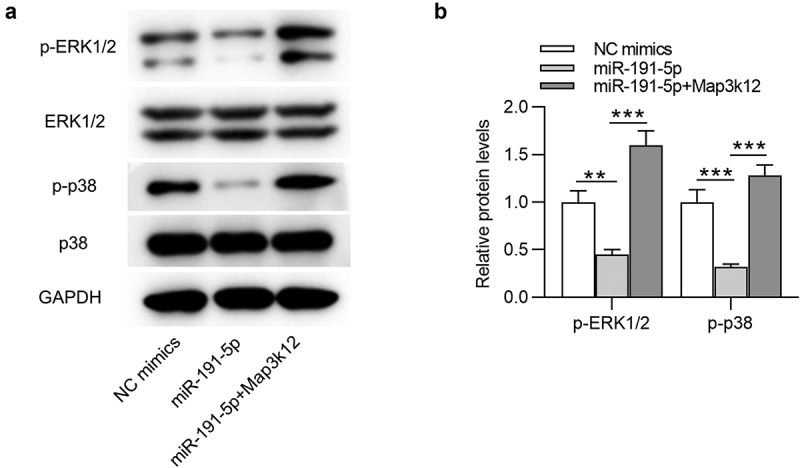
**MiR-191-5p inactivates the MAPK signaling by targeting Map3k12**. (a-b) Western blotting was performed to quantify protein levels of MAPK signaling-associated factors (ERK1/2, p-ERK1/2, p38, p-p38) in Aβ1-42-treated microglia transfected with NC mimics, miR-191-5p mimics or miR-191-5p mimics + pcDNA3.1/Map3k12. **p < 0.01, ***p < 0.001

As shown by Figure 5a-b, ERK and p38 protein expression at phosphorylated level (p-ERK1/2 and p-p38) were downregulated by miR-191-5p overexpression compared with those in the control mimics group. After co-transfection of miR-191-5p mimics and pcDNA3.1-Map3k12, the suppressive effect of miR-191-5p mimics on p-ERK1/2 and p-p38 protein levels in Aβ1-42-stimulated microglia was reversed (Figure 4a-b). Therefore, we concluded that miR-191-5p inactivates the MAPK signaling by targeting Map3k12 to downregulate Map3k12 expression.

## Discussion

AD is an aging-related neurodegenerative disease featured with cognitive dysfunction and neuron loss [43]. Available reports have suggested that Aβ cleaved from APP by β-secretase (BACE1) and γ-secretase plays a pivotal role in triggering complicated pathological cascades leading to AD [44]. The formation of amyloid plaque caused by Aβ extracellular accumulation is the hallmark of AD [45]. Mouse model of AD with Tau pathology or amyloid plaques shows hippocampal memory impairments [46]. APP/PS1 mice have been reported to establish classic mouse model of AD in various studies [47–49]. Here, pathological changes of hippocampus in APP/PS1 mice were evaluated by H&E staining and Nissl staining, which showed that APP/PS1 mice presented the loss of neurons and Nissl bodies in hippocampus. Moreover, high level of Aβ-positive granules in the hippocampal tissues of APP/PS1 mice was examined by immunohistochemical staining. All the results revealed that APP/PS1 mice in the study possess AD pathological features. Aβ accumulation induces apoptosis of neurons, which plays a pivotal role in AD development [50]. Hence, inhibiting apoptotic behavior of neurons is often considered as an approach for AD treatment [50]. Similar to previous studies [51], *in vitro* cell model of AD was established by using Aβ1-42 to treat microglial cells. In our study, Aβ1-42 stimulation decreased microglial cell viability and promoted microglial cell apoptosis, suggesting that Aβ1-42 induces microglial cell injury. Additionally, Aβ1-42 administration inhibited BACE1 and Tau-5 protein levels in microglial cells. BACE1 and Tau-5 is regarded as AD marker proteins in many studies focusing on AD investigation [52,53]. The above results indicated that *in vitro* cell model of AD was successfully established.

Multiple miRNAs have been proven to be participate in AD development, such as miRNA-101a [54], miRNA-483-5p [55], miRNA-455-3p [56] and miRNA-132 [57]. MiR-191-5p is a cluster of miR-191. MiR-191 downregulation elevates neuronal cell viability and decreases apoptosis of neurons, playing a protective role against isoflurane-induced neurotoxicity [58]. MiR-191 is related to the translational control mechanism involving hippocampal activity-depended processes, development, and differentiation [59]. MiR-191 presents significant lamina-specific level in human prefrontal cortex [60]. MiR-191-5p is poorly expressed in APP/PS1 mice [61]. In addition, genome-wide serum microRNA expression profiling identifies that miR-191-5p is downregulated in AD patients and miR-191-5p is one of serum biomarkers for AD [24]. Consistently, miR-191-5p is downregulated in hippocampal tissues of APP/PS1 mice in this study, suggesting that miR-191-5p may participate in AD development. Then, the effects of miR-191-5p on microglial cell injury were investigated. In cell model of AD, miR-191-5p overexpression attenuated Aβ1-42-induced microglial cell injury by elevating viability and reducing apoptosis rate of microglia. Additionally, miR-191-5p neutralized Aβ1-42-caused increase in BACE1 and Tau-5 protein levels in microglial cells.

Furthermore, Map3k12 was identified as the target gene of miR-191-5p in this study. Map3k12, also known as Dual leucine zipper kinase (DLK), can trigger neuronal stress response that modulates acute neuronal injury and neurodegeneration in models of chronic neurodegenerative diseases including Parkinson’s disease, AD and amyotrophic lateral sclerosis [62]. Pharmacological Map3k12 suppression relieves neuronal injury response and provides potent protection for neuronal cells against degeneration in response to neuronal insults [63,64]. Genetic Map3k12 deletion demonstrates benefits in mouse model of AD by inhibiting APP and Tau [64]. In this study, Map3k12 was negatively regulated by miR-191-5p in Aβ1-42-treated microglial cells. Moreover, all effects of miR-191-5p upregulation on the viability, apoptosis rate, BACE1 and Tau-5 protein levels in Aβ1-42-administrated microglia were offset by Map3k12 elevation. Thus, miR-191-5p mitigates microglial cell injury by targeting Map3k12.

Subsequently, the downstream signaling mediated by Map3k12 was investigated. Map3k12 (DLK) is a serine/threonine kinase that acts as an upstream activator of the MAPK pathways and plays a key role in cell differentiation, apoptosis and neuronal response to injury [65]. MAPKs are serine/threonine protein kinases that transmit extracellular signals to the nucleus and induce cell proliferation and differentiation [4,66]. The MAPK signaling is closely associated with AD neurodegeneration. p38 MAPK inhibitor can inhibit the activity of caspase-3-like and suppress cellular apoptosis [67]. Since the ERK1/2 pathway is engaged in cellular survival mechanisms, its activation attenuates cognitive impairments in AD [68]. Numerous studies have substantiated the involvement of the MAPK signaling in AD development. For example, Qingxin kaiqiao fang treatment restricts cellular apoptotic behaviors by regulating phosphorylation of p38 MAPK and ERK1/2, thus exerting neuroprotective effects on AD development [33]. MiR-132 inhibits oxidative stress and hippocampal inducible nitric oxide synthase (iNOS) expression by suppressing the MAPK signaling to alleviate cognitive deficits of rats with AD [32]. Herein, miR-191-5p overexpression inhibited protein levels of p-ERK1/2 and p-p38 in Aβ1-42-treated microglial cells, and the inhibitory effect on p-ERK1/2 and p-p38 was reversed by Map3k12 upregulation. The results suggested that miR-191-5p inactivates the MAPK pathway by targeting Map3k12 in microglia.

In conclusion, miR-191-5p alleviates Aβ1-42-induced microglia cell injury by targeting Map3k12 to inactivate the MAPK signaling, which may provide promising therapeutic biomarkers for AD treatment. In addition to the MAPK signaling, other pathways, such as the mTOR signaling [69] and the Wnt/β-catenin signaling [70], were reported to be associated with AD development. Studies on whether the miR-191-5p/Map3k12 axis can regulate these signaling pathways in AD pathogenesis will be carried out in the future. Moreover, further animal experiments will be designed to explore the signaling pathways *in vivo*.

## Conclusion

miR-191-5p is downregulated in the hippocampal tissues of APP/PS1 mice and Aβ1-42-treated microglial cells. Overexpressed miR-191-5p rescues the decrease in microglial cell viability and reverses the increase in cell apoptosis mediated by Aβ1-42 treatment. Additionally, miR-191-5p overexpression downregulates protein levels of AD markers (BACE1 and Tau-5) enhanced by Aβ1-42. Mechanistically, miR-191-5p targets Map3k12 to inactivate the MAPK signaling. Overall, miR-191-5p alleviates microglial cell injury by targeting Map3k12/MAPK signaling pathway.
